# Computational Design of a Thermo-Acidostable Endo-Polygalacturonase for Efficient Juice Extraction

**DOI:** 10.3390/foods15060980

**Published:** 2026-03-10

**Authors:** Zhong Cheng, Guobin Hou, Ting Zhang, Dongping Feng, Yanwen Zhang, Xingyue Wang, Liyan Yang, Maoyang Luo, Lixia Pan

**Affiliations:** 1 College of Food and Quality Engineering, Nanning University, University Engineering Research Center of High-Value Utilization of Tropical and Subtropical Specialty Fruits, Guangxi, Nanning 530200, China; zhongchengnu@163.com (Z.C.); 13387739234@163.com (G.H.); zhangting@unn.edu.cn (T.Z.); 18807705927@163.com (D.F.); luomaoyang03@163.com (M.L.); 2 State Key Laboratory of Non-food Biomass Energy Technology, Guangxi Academy of Sciences, Nanning 530007, China; 3 Guangxi Key Laboratory of Marine Natural Products and Combinatorial Biosynthesis Chemistry, Guangxi Academy of Marine Sciences, Nanning 530007, China

**Keywords:** protein engineering, endo-polygalacturonase, thermostability, MD simulations, juice extraction

## Abstract

The development of thermostable and pH-robust endo-polygalacturonases (endo-PGases) is crucial for industrial applications such as food processing. This study aimed to engineer the thermostability of an acidic, thermophilic endo-PGase (PoxaEnPG28B) by rigidifying its flexible regions. We employed an integrated computational strategy combining molecular dynamics (MD) simulations at elevated temperatures with in silico analyses of unfolding free-energy changes to identify and design stabilizing mutations. This approach successfully yielded the mutant D249K, which exhibited a 5 °C higher optimal temperature (70 °C) and a 68.8% longer half-life at 55 °C, and it retained over 76.8% activity at 75 °C. Notably, D249K maintained the wild-type’s optimal pH (5.0) and broad pH stability (3.0–8.0). Although it is not the absolute top performer in every single metric, D249K achieves the best overall balance between thermostability and pH robustness among all reported thermophilic endo-PGases. MD simulations revealed that its enhanced stability sems from reduced global and local flexibility and a more compact structure. In juice extraction applications, D249K increased yields by up to 98.5%, significantly surpassing the wild-type. This study demonstrates the efficacy of MD-guided flexible region engineering for the GH28 family and presents D249K as a highly promising industrial biocatalyst.

## 1. Introduction

Pectinase is a complex mixture of enzymes that can synergistically degrade pectin. It is extensively applied in food, agricultural, and textile industries because it degrades pectin to enhance material separation, rendering it a globally significant food enzyme preparation [1]. Endo-polygalacturonase (endo-PGase; EC 3.2.1.15)—a component of pectinase belonging to the glycoside hydrolase 28 (GH 28) family—catalyzes the random hydrolysis of α-1,4-glycosidic bonds in polygalacturonic acid chains. This cleavage mechanism confers the highest pectin degradation efficiency, rendering endo-PGase the most functionally critical element of pectinase systems [2]. The rapid development of food and agricultural industries also requires pectinase to be used under some special or even extreme conditions, such as the heat treatment of fruit pulp in juice extraction (60–65 °C), the heat treatment of sugar beet pulp in sugar production (70 °C), and high-temperature granulation processes in feed production (60–90 °C) [3]. However, most pectinases currently sold in the market—which contain endo-PGases as the main component—are sourced from *Aspergillus niger*. Despite *A. niger’s* GRAS (Generally Recognized As Safe) status, its endo-PGases exhibit low catalytic efficiency and limited thermostability, with functional activity typically declining above 50 °C [4,5].

Some thermophilic endo-PGases have been obtained from host strains through protein purification or cloning expression, such as the endo-PGase obtained from *Thermoascus aurantiacus* [6] and PG1 (a purified wild-type enzyme) obtained from *Penicillium occitanis* [7]. However, these traditional methods require screening target enzymes from massive protein databases or large microbial communities, which have disadvantages such as long time consumption, high cost, and large workload. With the rapid development of biotechnology, more efficient and mature enzymatic molecule modification techniques have been applied to the study of enzyme property enhancement [8]. Using this technology, enzymes with significantly improved catalytic properties have been obtained, such as alpha amylases, proteases, and lipases [9]. However, there are relatively few research reports on the modification of endo-PGases. Only Tu et al. optimized the surface charge of the endo-PGase (PG8fn) derived from *Achaetonium* sp. Xz8 using an enzyme thermal stabilization system (ETSS) [10], and they also studied the T3 loop region near the substrate channel of PG8fn [11]. Wang et al. modified the endo-PGase (*Tl*PGA) derived from *Talaromyces leycettanus* by introducing cysteine substitutions [12]. However, the optimal reaction temperature for PG8fn was only 55 °C, while *Tl*PGA only exhibited activity within a narrow pH range [13]. Therefore, it is still necessary to select high-performance endo-PGases for molecular modification in order to obtain new enzymes that meet industrial production needs and elucidate the relationship between important amino acids and functions.

Recent advances in computational power and sophisticated algorithms have accelerated protein engineering progress, enabling computational design platforms to successfully design enzymes with enhanced thermostability [8]. Among them, molecular dynamics (MD) simulation can guide protein design, contributing to structural stability by providing dynamic information [14]. Using MD to enhance the rigidity of flexible sites in enzymatic structures has become an important strategy for improving protein thermostability [15,16,17]. A highly stable enzyme is a key starting point in protein engineering, as it allows for the incorporation of various beneficial mutations while retaining its structural characteristics, thereby offering greater potential for functional enhancements through molecular engineering [18]. In our previous work, we identified PoxaEnPG28B from *Penicillium oxalicum* as an endo-PGase with promising acidophilic and thermophilic characteristics, marking it as a candidate with considerable application potential [19]. Nevertheless, similarly to other thermophilic enzymes, its practical utility is constrained by insufficient stability under prolonged heat stress.

To address this limitation, we targeted PoxaEnPG28B for comprehensive thermo-acidostability engineering. We employed a rational design pipeline utilizing MD simulations to identify global flexibility “hotspots,” followed by computational screening for stabilizing substitutions. This strategy resulted in the development of the D249K variant, which remarkably broke the common trade-off; in particular, it exhibited a significantly higher optimal temperature, extended half-life at challenging temperatures, and an exceptionally broad pH stability range, while also showing improved kinetic parameters. Its industrial superiority was unequivocally validated in a high-temperature juice extraction model. Our findings present D249K as a benchmark biocatalyst and demonstrate a targeted approach to designing multi-stress-resistant enzymes for demanding industrial applications.

## 2. Materials and Methods

### 2.1. Modeling and Molecular Dynamics Simulation

The three-dimensional structure model of PoxaEnPG28B for the wild-type (WT) enzyme and its mutants was generated using AlphaFold2 [20] and visualized with PyMol 2.4 [21]. MD simulations were conducted using AMBER 22 with the AMBER ff14SB force field [22]. Each enzyme structure was placed in an octahedral simulation box filled with TIP3P water molecules, with Na^+^ and Cl^−^ ions added to neutralize charges and simulate physiological ionic strength. Prior to production simulations, systems were minimized using the steepest descent and conjugate gradient methods. This was followed by 100 ps of isothermal–isovolumetric (NVT) simulations to equilibrate system temperature and 100 ps of isothermal–isobaric (NPT) simulations to equilibrate the system pressure to 1 bar.

Production MD simulations were conducted at study-specific temperatures. The WT enzyme was simulated at 323 K and 338 K, with 338 K as thermal stress to probe temperature-induced conformational responses. The D249K mutant was modeled at 323 K for direct, controlled comparisons with WT under identical thermal conditions. All simulations ran for 50 ns under periodic boundary conditions with a 2 fs time step. Post-simulation analyses focused on 323 K WT/D249K comparisons, including calculations of the root mean square deviation (RMSD), root mean square fluctuation (RMSF), radius of gyration (Rg), and solvent-accessible surface area (SASA) [23].

### 2.2. Strains, Plasmids, and Chemicals

Gene cloning was performed using *Escherichia coli* DH5α (Sangon Biotech, Shanghai, China) as the host strain. Heterologous expression of PoxaEnPG28B and its mutants was carried out using the plasmid pPIC9K in *Pichia pastori* GS115. The high-fidelity enzyme 2× Phanta Max Master Mix was purchased from Vazyme (Nanjing, China). The restriction endonuclease *Sac*I and protein marker were purchased from Takara (Dalian, China) and TransGen Biotech (Beijing, China), respectively. D-galacturonic acid and polygalacturonic acid (PGA) were acquired from Beijing Solarbio Science & Technology (Beijing, China). Endo-β-N-acetylglucosaminidase H (Endo H) was procured from New England Biolabs (Beijing, China). The TIANprep Mini Plasmid Kit was obtained from Tiangen Biotech (Beijing, China). All other chemicals were of analytical grade and commercially available.

### 2.3. Site-Directed Mutagenesis, Expression, and Purification

Mutant DNA sequences without signal peptides were synthesized and cloned into the expression vector pPIC9K by Nanning Jienisi biotechnology (Nanning, China). The recombinant plasmids were linearized with *Sac*I and then individually transformed into competent *Pichia pastoris* GS115 cells using electroporation. Recombinant WT and mutant enzymes were expressed and purified following a protocol described previously [24]. The purity and molecular weight of recombinant proteins were determined via sodium dodecyl sulfate–polyacrylamide gel electrophoresis (SDS-PAGE). Purified proteins were deglycosylated using Endo H and then analyzed via SDS-PAGE.

### 2.4. Enzyme Characterization

Polygalacturonase activity was measured using the previously reported 3,5-dinitrosalicylic acid (DNS) method [25]. One unit of polygalacturonase activity was defined as the amount of enzyme that releases 1 µmol of D-galacturonic acid per minute in the catalytic system using PGA as the substrate under the specified assay conditions. The protein concentration was determined using the Bradford method using bovine serum albumin as a standard sample [26].

Optimum temperatures were determined by measuring enzymatic activity after 15 min incubations across a temperature gradient in a pH 5.0 buffer. To measure thermostability, the enzyme solutions were incubated at 50 and 55 °C for 1 h without the substrate, and residual activities were measured every 15 min under standard conditions. The preliminary screening of mutants was carried out using the methods described above, and the mutant with the optimal temperature performance was used for further research.

The optimal pH of the purified endo-PGases was determined by measuring enzyme activity at 50 °C and pH values ranging from 3.0 to 7.0. The pH stability of the enzyme was evaluated as follows: the enzyme solution was incubated at 25 °C in buffer solutions with varying pH values (0.1 M Citrate-Na_2_HPO_4_ buffer for pH 3.0–7.0 and 0.1 M Tris-HCl buffer for pH 7.0–9.0) for 24 h (without substrate addition), and the residual enzyme activity was then measured.

In the study of optimal reaction temperatures and optimal reaction pH values, the highest enzyme activity was defined as 100%, and other enzyme activities were calculated as relative values. In the study of temperature stability and pH stability, the untreated enzyme activity was defined as 100%, and all residual enzyme activities were calculated as relative values.

### 2.5. Kinetic Parameter Determination

To measure the kinetic characteristics of the WT and its optimal mutant, enzyme activities were determined under standard conditions with different concentrations (0.1–1.5%) of PGA; then, the *K*_m_ and *V*_max_ values were calculated using the Lineweaver–Burk method. The half-life of an enzyme (*t*_1/2_) at 55 °C was determined by measuring the residual enzyme activity at different incubation times under standard conditions.

### 2.6. Molecular Docking and Mechanism Analysis

To elucidate the structural basis underlying the enhanced thermostability of the mutant compared with the WT, molecular docking analysis was performed. The enzyme–substrate complexes were modeled using the HDOCK server (access on 26 July 2022) [27]. The binding pose with the lowest binding free energy and a physically reasonable orientation was selected for subsequent structural and interaction analyses.

### 2.7. Enzymatic Treatment of Fruit Pulp

Three fruits—Orah (a hybrid citrus variety), plantain, and papaya—were obtained from a local market. The fruits were peeled and deseeded, then blended with an equal weight (1:1, *w*/*w*) of distilled water to form a homogeneous pulp. For enzymatic treatment, 25 g of pulp was incubated at 65 °C for 15 min with the enzyme at a dosage of 0.04 mg protein/kg pulp. The mixture was subsequently centrifuged at 2370× *g* for 10 min to remove solids. The supernatant was collected, and its parameters were analyzed according to established methods [25]. The pulps without enzyme addition were subjected to the same processing and served as the control.

### 2.8. Statistical Analysis

All enzymatic experiments were performed in triplicate or more. Data analysis was conducted using the Origin 9.0 software (Origin Lab, Northampton, MA, USA).

## 3. Results and Discussion

### 3.1. Design the Thermostable Mutants of PoxaEnPG28B

The balance between rigid and flexible regions govern protein folding kinetics and structural integrity. Due to the weak non-covalent interactions between amino acid residues, the flexible region is often the earliest deconstructed region in the protein structure when the temperature increases [28]. Therefore, enhancing the rigidity of flexible protein regions can hinder the protein unfolding process induced by elevated temperatures, thereby improving the protein’s thermal tolerance. In recent years, the strategy of flexible site modification has been successfully applied to the thermostability modification of enzymes due to the following advantages: strong universality, high mutation efficiency, low loss of enzyme activity, and multifunctional synergy [29,30,31]. However, there are few reports on its application in the GH 28 family.

**Figure 1:**
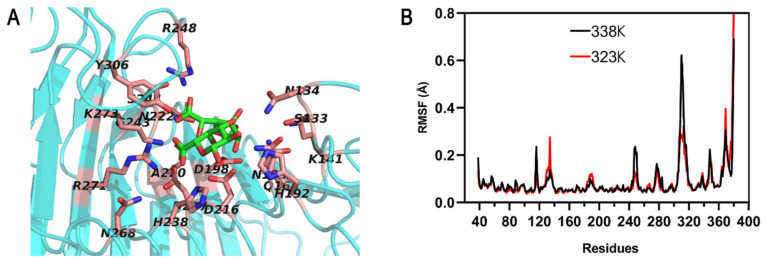
Predicted 3D structural model and MD simulations of PoxaEnPG28B. (**A**) Amino acid residues within a 5 Å radius of the active site are shown in light red, and digalacturonic acid is shown in green. (**B**) Residue-wise RMSF profiles from MD simulations conducted at 323 K and 338 K.

Based on the above, this study aims to enhance the thermostability of the previously obtained endo-PGase (PoxaEnPG28B) with acidophilic and thermophilic characteristics by rigidifying its flexible regions through protein engineering. We first used AlphaFold2 to predict the three-dimensional structure of PoxaEnPG28B. The results indicated that it shared similar structural features with another GH 28 family, which contained 10 complete parallel β-helices, each comprising three or four β-sheets (Supplementary Figure S1) [3]. However, compared to these family members, this enzyme possesses a redundant sequence (Ser20–Thr38) following the signal peptide (Met1–Ala19)—a feature that has not been reported for the N-terminal region of other endo-PGases. To further investigate the effect of this sequence on the thermostability of PoxaEnPG28B, we constructed a mutant (NSD, N-terminal sequence deletion) that removed this redundant sequence for enzymatic property analyses. Additionally, we also identified the amino acid residues located within the active center (i.e., the structural region within 5 Å of the substrate) (Figure 1A).

MD simulations of PoxaEnPG28B were performed at 323 K and 338 K, followed by root mean square fluctuation (RMSF) analysis (Figure 1B). The amino acid residues with RMSF values greater than 0.3 Å were defined as potential flexible sites. By integrating these results with prior primary structure comparisons (excluding the conserved residues and amino acids within the 5 Å range of the active center) [19], ten highly flexible amino acid sites were identified (Table 1). Suitable substitution residues for these ten sites were then determined via computational analyses of unfolding free-energy changes (ΔΔG; http://protein.org.cn/ddg.html; accessed on 9 August 2022) [32], where a positive ΔΔG (kcal/mol) indicates increased stability.

Then, the mutant DNA sequences without signal peptides were synthesized and cloned into the expression vector pPIC9K, and 32 recombinants (including the WT and NSD) were finally obtained. After plasmid linearization and electroporation of *P. pastoris* GS115, 30 recombinant proteins (except for the mutants of T38Q and Y367W) were successfully expressed. Then, the recombinant proteins were purified using a straightforward one-step ultrafiltration method [24]. The SDS-PAGE results of purified proteins showed two bands around approximately 40.0 kDa (Supplementary Figure S2). Following Endo H treatment, the de-glycosylated recombinant proteins showed a distinct band, which matched the theoretical value of 38.0 kDa [19]. This finding corroborates previous observations that heterologous proteins expressed in *P. pastoris* are prone to excessive N-glycosylation [24].

In order to screen mutants with high-temperature performance, the optimal temperature of each recombinant enzyme was determined (Table 1). It was found that the site-directed mutants of the predicted flexible residues (G309, D310, and S317) and the NSD showed a reduced optimal temperature relative to the WT. Among all mutants, only D249K exhibited an elevated optimal temperature, while other mutants retained the WT’s optimal temperature.

After incubating the WT and mutant enzymes at 50 and 55 °C for 1 h in the absence of the substrate, the residual activities were measured under standard assay conditions. As summarized in Table 2, several mutants, including T38K, T38F, D249Q, D249E, D249K, D249S, D314K, and D314T, demonstrated strong thermostability at 55 °C, with residual activities exceeding 70%. A positive correlation was observed for mutants with reduced optimal temperatures, which concurrently exhibited poor thermal stability. For all other mutants, thermostability remained largely unchanged relative to the WT.

The NSD mutant exhibited poor temperature characteristics, indicating that this redundant sequence may play an important role in the thermostability of PoxaEnPG28B. Previous studies have reported that the thermostability of some enzymes decreased after truncation of their amino-terminal sequences, mainly due to the disruption of hydrogen bond networks [33] or the increased flexibility of local structures [34]. Despite the established positive correlation between enzymatic optimal temperature and thermostability, the two parameters (optimal temperature and thermostability) exhibit non-parallel scaling under thermal selection pressures due to distinct structural constraints. It is possible that the adaptation of enzymes to different environmental temperatures requires a balance between structural stability and conformational flexibility [35].

### 3.2. Determination of Kinetic Parameters and Enzymatic Properties of WT and Variants

**Figure 2:**
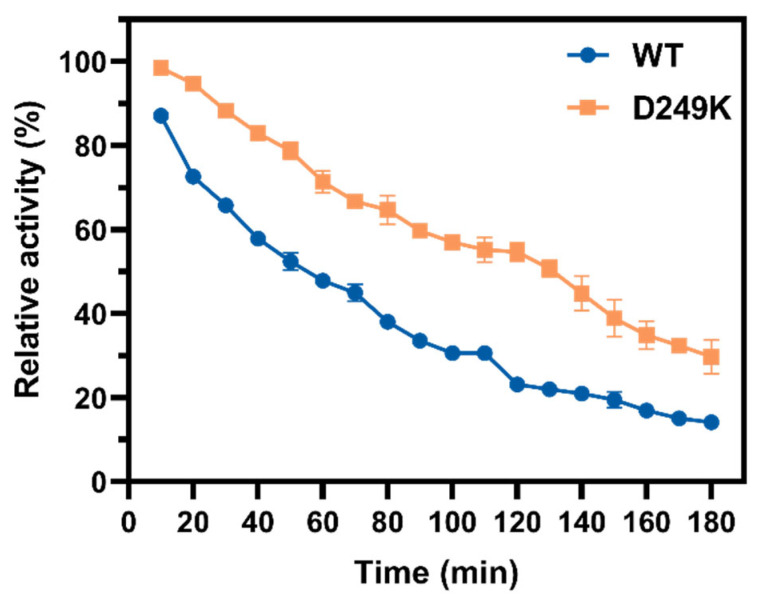
Comparative thermostability of the WT enzyme and the D249K mutant at 55 °C.

Among all variants, only the D249K mutant showed significant improvement over the WT, with a 5 °C increase in optimal temperature and a 26.2% higher residual activity at 55 °C. Furthermore, its half-life (*t*_1/2_) at 55 °C was determined to be 108 min, which is 68.8% longer than that of the WT (64 min) (Figure 2). These enhancements were achieved through a rational design strategy integrating MD simulations to identify flexible sites with computational analyses of unfolding free-energy changes. This integrated approach successfully yielded a D249K mutant with improved thermostability, demonstrating the applicability of this method for engineering enzymes within the GH28 family. This methodology aligns with previous successful cases. For example, Feng et al. utilized MD simulations to modify the flexible N-terminal loop of *Bacillus subtilis* L-Asparaginase, obtaining a double variant with significantly enhanced specific activity and thermostability [36]. Similarly, combined homology modeling and MD simulations resulted in improved thermostability in a methyl parathion hydrolase [37].

To assess the impact on catalytic properties, we determined the kinetic parameters of the enzymes on PGA (Supplementary Figure S3). As shown in Table 3, D249K (89,285 U/mg) exhibited a higher *V*_max_ than the WT (70,921 U/mg), although its *K*_m_ (2.7 mg/mL) was slightly higher than that of the WT (2.6 mg/mL). Consequently, the catalytic efficiency (*K*_cat_/*K*_m_) of D249K (21,197 mL/(s·mg)) was improved relative to the WT (17,485 mL/(s·mg)). The elevated *K*_m_ value suggested that the mutation, despite being distant from the active site, reduced substrate affinity; likely by introducing structural rigidity. Conversely, the improved catalytic efficiency indicated that this increased rigidity more effectively stabilized the transition state, thereby increasing the turnover rate [38].

Because endo-PGase is a critically important pectinase in the food, agricultural, and livestock industries, research to discover variants with superior properties has continually been conducted [1]. As summarized in Table 4, recently reported endo-PGases predominantly originate from filamentous fungi, consistent with the known taxonomic source preference where bacterial producers are rare. Among these, several exhibited thermophilic traits, including *Tl*PGA from *Talaromyces leycettanus* (70 °C) [13], pePGA from *Penicillium rolfsii* (60 °C) [39], and *Ml*PG28B from *Mucor lusitanicus* (60 °C) [40]. Among these, D249K and *Tl*PGA stood out, sharing the highest optimum temperature (70 °C), which is among the highest reported for this enzyme class. Notably, D249K demonstrated superior thermal robustness by retaining 76.8% of its relative activity even at 75 °C (Supplementary Figure S4). This exceptional maintenance of activity under supra-optimal temperatures underscores the significant industrial application value of D249K, particularly for high-temperature bioprocessing.

Modern industrial production not only requires enzymes to exhibit high efficiency and thermostability but also to be functionally robust across a broad pH range. However, some enzymes may change their optimal reaction pH value after thermal stabilization modification. For example, with respect to the wild-type glucose isomerase TK4GI from *Geobacillus caldoxylosilyticus*, its optimal reaction pH was 7.5, but the optimal reaction pH values for mutants H99Q, V184T, and D102N after thermostability modification were 6.0, 6.5, and 6.0, respectively [45]. As another example, the wild-type phosphatase wtPAP exhibited catalytic efficiency at pH 5.5, but the mutant mutPAP with improved thermostability showed the highest catalytic efficiency at pH 5.0 [46].

**Figure 3:**
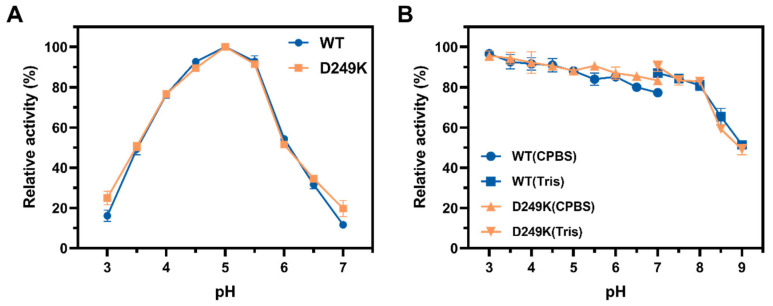
pH optimum and stability profiles of the WT and the D249K mutant. (**A**) Optimal pH for enzyme activity. (**B**) Residual activity after incubation in buffers of different pH values. The buffers used were 0.1 M Citrate-Phosphate (CPBS) and Tris-HCl. Error bars indicate the standard deviation of triplicate measurements.

In this study, to further verify whether the pH activity of the mutated enzyme was affected by thermostability modification, we measured the optimal reaction pH value and pH stability range of the enzyme using the WT as a control (Figure 3). The results showed that the D249K mutant, similarly to the WT, had an optimal pH value of 5.0 and is a typical acidic enzyme. Remarkably, this mutant exhibited a broad pH stability range from 3.0 to 8.0—notably broader than those of several recently reported endo-PGases, including PoxaEnPG28C (pH 3.0–6.5) [41], pePGA (pH 3.5–8.0) [39], PgaB (pH 4.5) [42], PG II (pH 3.0–7.0) [43], and PGase (pH 4.0–5.0) [44]. Although its pH range is narrower than that of *Ml*PG28B [40], D249K surpasses the latter in thermal stability. Furthermore, D249K maintained high activity across nearly the entire acidic pH scale, a range wider than that of *Tl*PGA [13]. Therefore, D249K stands out among reported thermophilic endo-PGases by uniquely combining robust thermal stability with broad pH stability, positioning it as one of the most effective endo-polygalacturonases. This combination of traits directly addresses key industrial needs, as pectinases are predominantly used in acidic environments—such as fruit processing and animal feed production—that favor enzymes with broad pH activity and stability. D249K meets these demands by offering a higher optimal temperature, improved thermostability, and boosted catalytic efficiency while fully retaining the wide pH adaptability of the wild-type enzyme. Such a profile renders it a highly promising industrial biocatalyst.

### 3.3. Mechanism of Enhanced Thermostability

**Figure 4:**
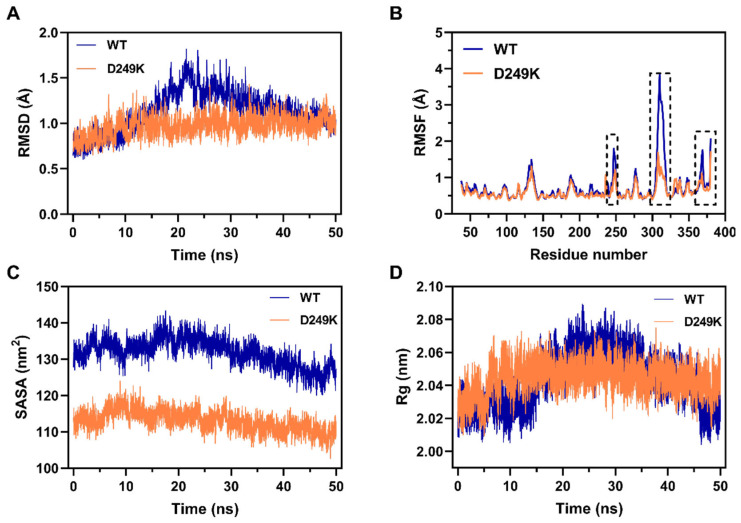
Molecular dynamics (MD) simulation of the WT and D249K variant at 323 K. (**A**) Backbone RMSD during the 50 ns simulation, reflecting global structural stability. (**B**) Per-residue RMSF, with dashed boxes marking regions where D249K shows notably reduced flexibility. (**C**) SASA over time, where lower values in D249K indicate a more compact structure. (**D**) Time evolution of the radius of gyration (Rg), representing overall structural compactness.

To elucidate the molecular mechanisms underlying the improved thermostability of the D249K mutant, all-atom MD simulations were performed for both the WT and D249K at 323 K. The RMSD metrics exhibited no systematic drift over the 50 ns simulation period, confirming that a stable sampling regime was maintained. During the 50 ns simulations, the WT exhibited pronounced backbone fluctuations, with RMSD values varying between 0.60 and 1.80 Å, whereas D249K maintained a markedly more constrained conformational ensemble, with RMSD values ranging from 0.63 to 1.43 Å and an average RMSD of approximately 0.97 Å throughout the trajectory (Figure 4A). Since RMSD reflects the collective motion of the protein backbone, the reduced fluctuation amplitude and fewer transitions between conformational states in D249K indicate a more structurally restrained and thermally resilient fold.

Consistent with the RMSD results, RMSF analyses revealed a substantial reduction in local flexibility in several thermally sensitive regions of the mutant. Three segments—Region I (residues 239–255), Region II (residues 301–320), and Region III (residues 364–380)—exhibited markedly lower residue-level fluctuations relative to the WT (Figure 4B). These regions are located within (or adjacent to) structural elements that contribute to the global fold; therefore, their reduced mobility likely suppresses local unfolding events that typically precede global thermal destabilization. The attenuation of local dynamics thus contributes to an overall increase in structural rigidity.

To further assess the compactness of the protein architecture, the solvent-accessible surface area (SASA) and radius of gyration (Rg) were evaluated. The D249K variant exhibited a significantly lower average SASA (113.01 ± 0.81 nm^2^) compared with the WT (131.88 ± 1.42 nm^2^) (Figure 4C), indicating reduced solvent exposure of hydrophobic core residues. Such compaction is commonly associated with enhanced thermostability, as it lowers the entropic propensity toward thermal unfolding and favors the retention of the folded state under heat stress. Likewise, the Rg of D249K remained consistently stable (2.04 ± 0.01 nm) with minimal conformational dispersion, whereas the WT exhibited larger fluctuations (2.04 ± 0.03 nm) over the simulation (Figure 4D). Statistical analyses confirmed that this difference is significant (*p* < 0.05), supporting the conclusion that the mutant preserves a more compact and cohesive tertiary structure.

Taken together, the MD results demonstrate that the enhanced thermostability of D249K arises from the synergistic effects of reduced global and local structural flexibility, increased packing compactness, and a more conformationally constrained structural ensemble. These features collectively strengthen its resistance to temperature-induced unfolding, enabling the mutant to maintain structural integrity at elevated temperatures more effectively than the WT protein.

### 3.4. Enzymatic Extraction of Fruit Juice

In order to evaluate the application effects of D249K and WT in juice production, juice extraction experiments were conducted on three types of fruits. In the experiment, it was observed that, without enzyme addition, the juice yields of orah, plantain, and papaya were 17.8 g, 8.6 g, and 15.3 g, respectively; the viscosities were 2.5 mPas, 5.2 mPas, and 2.2 mPas, respectively; and the transmittances were 14.4%, 38.3%, and 49.2%, respectively. Upon the addition of D249K and WT, marked improvements in the relevant parameters were observed (Table 5). Overall, the application performance of D249K was superior to that of the WT. Among the evaluated metrics, juice yield proved to be the most critical factor. Following enzymatic treatment, D249K increased the juice yield of orah, plantain, and papaya by 10.8%, 98.5%, and 22.3%, respectively, significantly outperforming the WT yield increments of 7.4%, 86.4%, and 19.8%. These results indicate that D249K is markedly more effective than WT in the context of high-temperature juice extraction.

## 4. Conclusions

In conclusion, this study successfully engineered a thermostable acidic endo-polygalacturonase (PoxaEnPG28B) through a rational, computation-guided strategy focused on rigidifying flexible regions. The integrative approach—combining molecular dynamics simulations to identify dynamic “hotspots” with in silico stability prediction—proved to be highly effective for the GH28 family, culminating in the superior mutant D249K. This variant sets a new benchmark by combining an elevated optimum temperature of 70 °C, significantly enhanced thermostability (68.8% longer half-life), and broad pH stability (3.0–8.0) without compromise. Molecular dynamics simulations revealed that these improvements originate from a more rigid and compact conformational ensemble with reduced solvent-accessible surface area. Crucially, the D249K mutant also exhibited superior catalytic efficiency and demonstrated significantly improved efficacy in high-temperature fruit juice extraction, increasing yields by up to 98.5%. Our work not only delivers a potent industrial biocatalyst, but also validates a generalizable framework for the thermostability engineering of pectinases and related enzymes.

## Figures and Tables

**Table 1:** The mutants and their corresponding optimal temperatures.

Name	Optimal Temperature (°C)	Name	Optimal Temperature (°C)
WT	65	D310K	50
NSD	60	D310M	45
T38C	65	D310F	50
T38I	65	D310T	45
T38K	65	D310W	45
T38F	65	D310V	45
D249Q	65	H312K	65
D249E	65	D314N	65
D249K	70	D314Q	65
D249S	65	D314E	65
D250H	65	D314I	65
G309Q	60	D314K	65
G309E	60	D314T	65
D310I	45	A315P	65
D310L	45	S317I	60

**Table 2:** Thermostability of WT and mutated enzymes at 50 °C and 55 °C.

Name	50 °C Residual Activity (%)	55 °C Residual Activity (%)	Name	50 °C Residual Activity (%)	55 °C Residual Activity (%)
WT	90.2 ± 0.27	47.4 ± 1.30	D310K	89.2 ± 0.3	1.27 ± 0.6
NSD	86.9 ± 0.39	28.3 ± 0.73	D310M	67.5 ± 1.17	0.72 ± 0.36
T38C	98.8 ± 1.64	68.6 ± 1.78	D310F	84.9 ± 1.1	3.3 ± 0.26
T38I	97.4 ± 1.11	65.4 ± 1.52	D310T	69.2 ± 1.12	2.1 ± 3.24
T38K	97.9 ± 1.68	78.4 ± 1.03	D310W	49.9 ± 1.42	0.81 ± 1.08
T38F	98.2 ± 0.23	76.5 ± 1.43	D310V	46.6 ± 1.17	1.5 ± 0.19
D249Q	98.9 ± 0.64	70.8 ± 1.94	H312K	98.7 ± 1.46	65.6 ± 0.91
D249E	98.8 ± 0.93	73.3 ± 0.72	D314N	97.9 ± 1.20	59.8 ± 0.54
D249K	96.1 ± 1.11	73.6 ± 1.20	D314Q	84.1 ± 0.83	66.3 ± 0.88
D249S	97.1 ± 0.52	72.9 ± 0.71	D314E	97.9 ± 0.88	64.7 ± 1.15
D250H	99.2 ± 0.76	27.6 ± 1.13	D314I	91.7 ± 0.43	57.4 ± 1.27
G309Q	94.2 ± 0.48	30.2 ± 1.02	D314K	95.6 ± 1.16	71.5 ± 1.40
G309E	87.2 ± 0.80	32.3 ± 0.45	D314T	90.3 ± 1.99	70.4 ± 2.48
D310I	64.3 ± 0.44	2.3 ± 0.38	A315P	94.6 ± 0.76	57.9 ± 0.89
D310L	64.4 ± 0.86	1.6 ± 0.25	S317I	90.5 ± 0.38	20.3 ± 0.33

**Table 3:** The kinetic characteristics of WT and mutant D249K.

	*V*_max_ U/mg	*K*_m_ mg/mL	*K*_cat_ 1/s	*K*_cat_/*K*_m_ mL/(s·mg)
D249K	89,285	2.7	57,233	21,197
WT	70,921	2.6	45,462	17,485

**Table 4:** The main enzymatic characteristics of endo-PGases reported in recent years.

Name	Temperature Optimum (°C)	pH Optimum	Thermostability (°C)	pH Stability	Reference
D249K	70 °C ^a^	5.0	~74% at 55 °C for 60 min	3.0–8.0	This study
WT	65 °C ^a^	5.0	~47% at 55 °C for 60 min	3.0–8.0	[19]
PoxaEnPG28C	45 °C ^a^	4.5	~20% at 55 °C for 60 min	3.0–6.5	[41]
*Tl*PGA	70 °C ^b^	3.5	~100% at 55 °C for 15 min	ND	[13]
pePGA	60 °C ^b^	6.0	~70% at 45 °C for 60 min	3.5–8.0	[39]
PgaB	40 °C ^c^	4.5	~90% at 30 °C for 420 min	4.0–5.0	[42]
PG II	45 °C ^a^	3.5–4.5	ND	3.0–7.0	[43]
*Ml*PG28B	60 °C ^d^	5.0	~40% at 70 °C for 60 min	3.0–11.0	[40]
PGase	40 °C ^e^	4.5	~76% at 50 °C for 60 min	4.0–5.0	[44]

^a–e^. The reaction times were 15 min ^a^, 10 min ^b^, 20 min ^c^, 30 min ^d^, and 5 min ^e^, respectively. ND: Not determined.

**Table 5:** Effects of enzymes on the fruit juice production.

Fruits	WT			D249K		
	Increment of Yield (%)	Reduction in Viscosity (%)	Increment of Light Transmittance (%)	Increment of Yield (%)	Reduction in Viscosity (%)	Increment of Light Transmittance (%)
Orah	7.4 ± 2.2	12.3 ± 2.2	15.3 ± 1.7	10.8 ± 3.1	18.7 ± 2.6	19.7 ± 2.4
Plantain	86.4 ± 3.5	67.1 ± 2.8	40.3 ± 2.1	98.5 ± 4.3	73.5 ± 3.2	46.6 ± 1.6
Papaya	19.8 ± 1.1	24.5 ± 2.9	37.7 ± 1.7	22.3 ± 3.5	28.6 ± 1.2	42.7 ± 0.9

## Data Availability

Data will be made available upon request.

## Supplementary Materials

The following supporting information can be downloaded at: https://www.mdpi.com/article/10.3390/foods15060980/s1. Figure S1:The structure model of PoxaEnPG28B generated by AlphaFold2. The α-helix, β-sheets and other structural regions (including loops) are indicated in blue, yellow and gray, respectively; Figure S2: SDS-PAGE analysis of purified recombinants. Lanes: WT-1 and D249K-1, purified recombinant WT and D249K mutant by one-step ultrafiltration method; WT-2 and D249K-2, WT and D249K mutant after N-deglycosylation with Endo H; Figure S3: Michaelis-Menten constant of WT (A) and D249K (B). The kinetic parameters were determined by assaying the reaction rates for polygalacturonic acid at concentrations ranging from 1.0–15.0 g/l under the standard assay conditions. The Michaelis-Menten constant (*K*_m_) and the maximum reaction velocity (*V*_max_) were obtained from the Lineweaver-Burk plot; Figure S4: Effects of temperatures on the recombinant enzymes activities measured in Citrate-Phosphate (CPBS) buffer (pH 5.0) for 15 min.
