# Supplementary material for: Computational Design of a Thermo-Acidostable Endo-Polygalacturonase for Efficient Juice Extraction
Source: Foods. 2026 Mar 10;15(6):980. doi: 10.3390/foods15060980 (PMC13025290; doi:10.3390/foods15060980)
Supplement: Supplementary file 1 [file foods-15-00980-s001.zip › foods-4168298-supplementary.pdf]

Journal: **Foods**

Supplementary material for

Computational Design of a Thermo-Acidostable Endo-Polygalacturonase for Efficient Juice Extraction

Zhong Cheng [1], Guobin Hou [1], Ting Zhang [1], Dongping Feng [1], Yanwen Zhang [1], Xingyue Wang [2,3], Liyan Yang [2,3], Maoyang Luo [1] and Lixia Pan [2,3,*]

[1] College of Food and Quality Engineering, Nanning University, University Engineering Research Center of High-Value Utilization of Tropical and Subtropical Specialty Fruits, Guangxi, Nanning 530200, China;
zhongchengnu@163.com (Z.C.); 13387739234@163.com (G.H.); zhangting@unn.edu.cn (T.Z.); 18807705927@163.com (D.F.); zhangyanwen@unn.edu.cn (Y.Z.); luomaoyang03@163.com (M.L.)

[2] State Key Laboratory of Non-food Biomass Energy Technology, Guangxi Academy of Sciences, Nanning 530007, China; w555hmbb@163.com (X.W.); yangliyan.1988@163.com (L.Y.)

[3] Guangxi Key Laboratory of Marine Natural Products and Combinatorial Biosynthesis Chemistry, Guangxi Academy of Marine Sciences, Nanning 530007, China

* Correspondence: panlixia@gxas.cn; Tel.: +86-771-2503970; Fax: +86-771-2503980

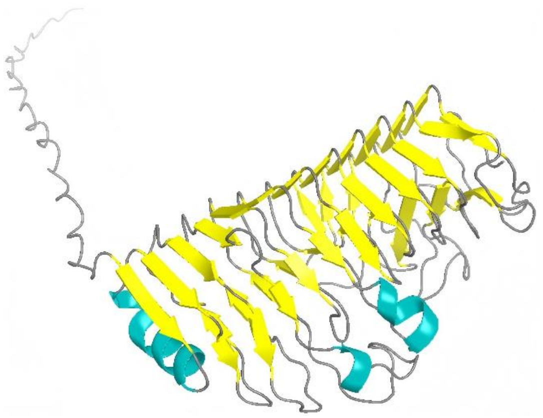

Figure S1: The structure model of PoxaEnPG28B generated by AlphaFold2. The α-helix, β-sheets and other structural regions (including loops) are indicated in blue, yellow and gray, respectively.

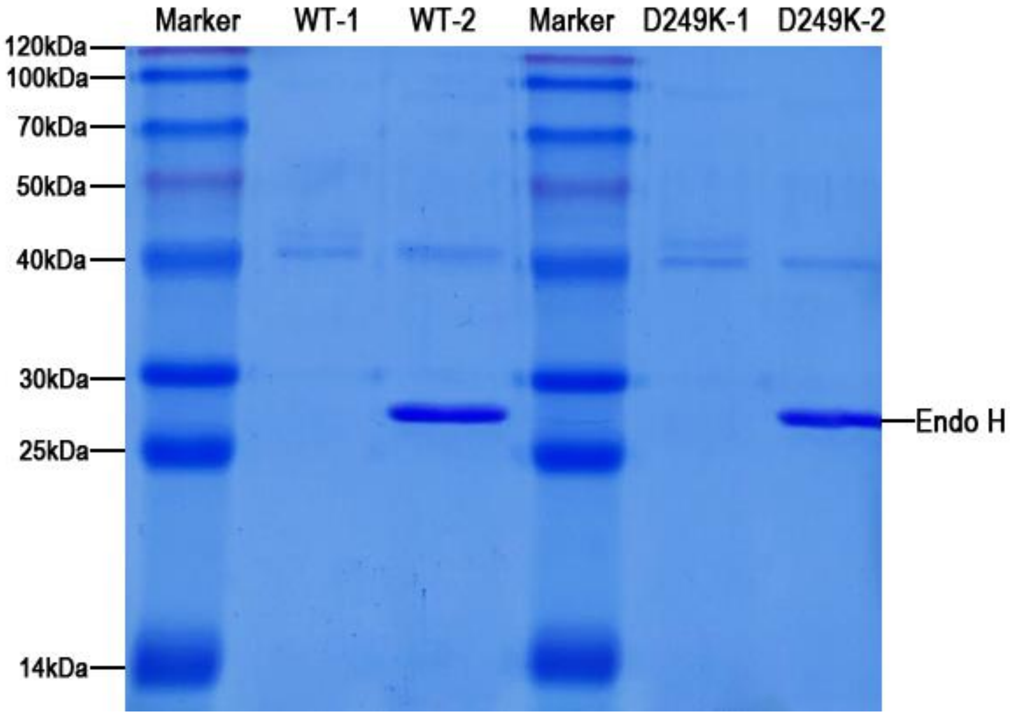

Figure S2: SDS-PAGE analysis of purified recombinants. Lanes: WT-1 and D249K-1, purified recombinant WT and D249K mutant by one-step ultrafiltration method; WT-2 and D249K-2, WT and D249K mutant after N-deglycosylation with Endo H.

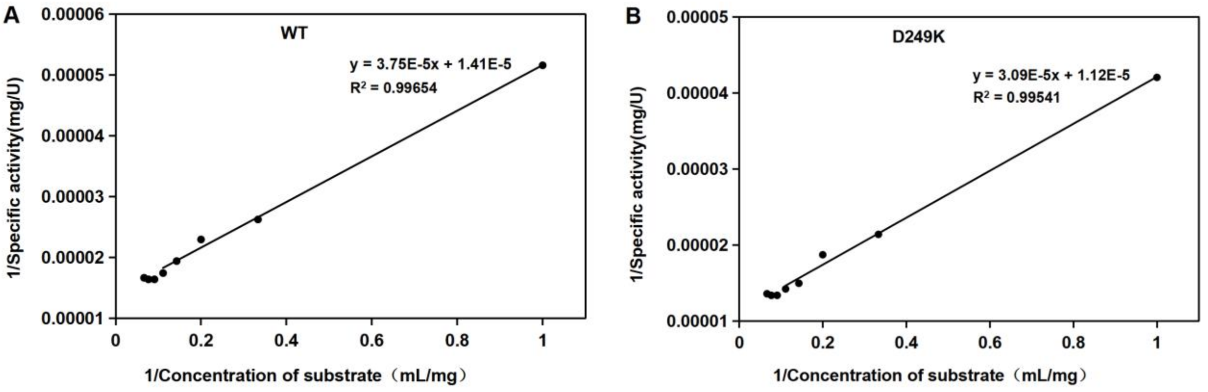

Figure S3: Michaelis-Menten constant of WT (A) and D249K (B). The kinetic parameters were determined by assaying the reaction rates for polygalacturonic acid at concentrations ranging from 1.0–15.0 g/l under the standard assay conditions. The Michaelis-Menten constant (K_m) and the maximum reaction velocity (V_{max}) were obtained from the Lineweaver-Burk plot.

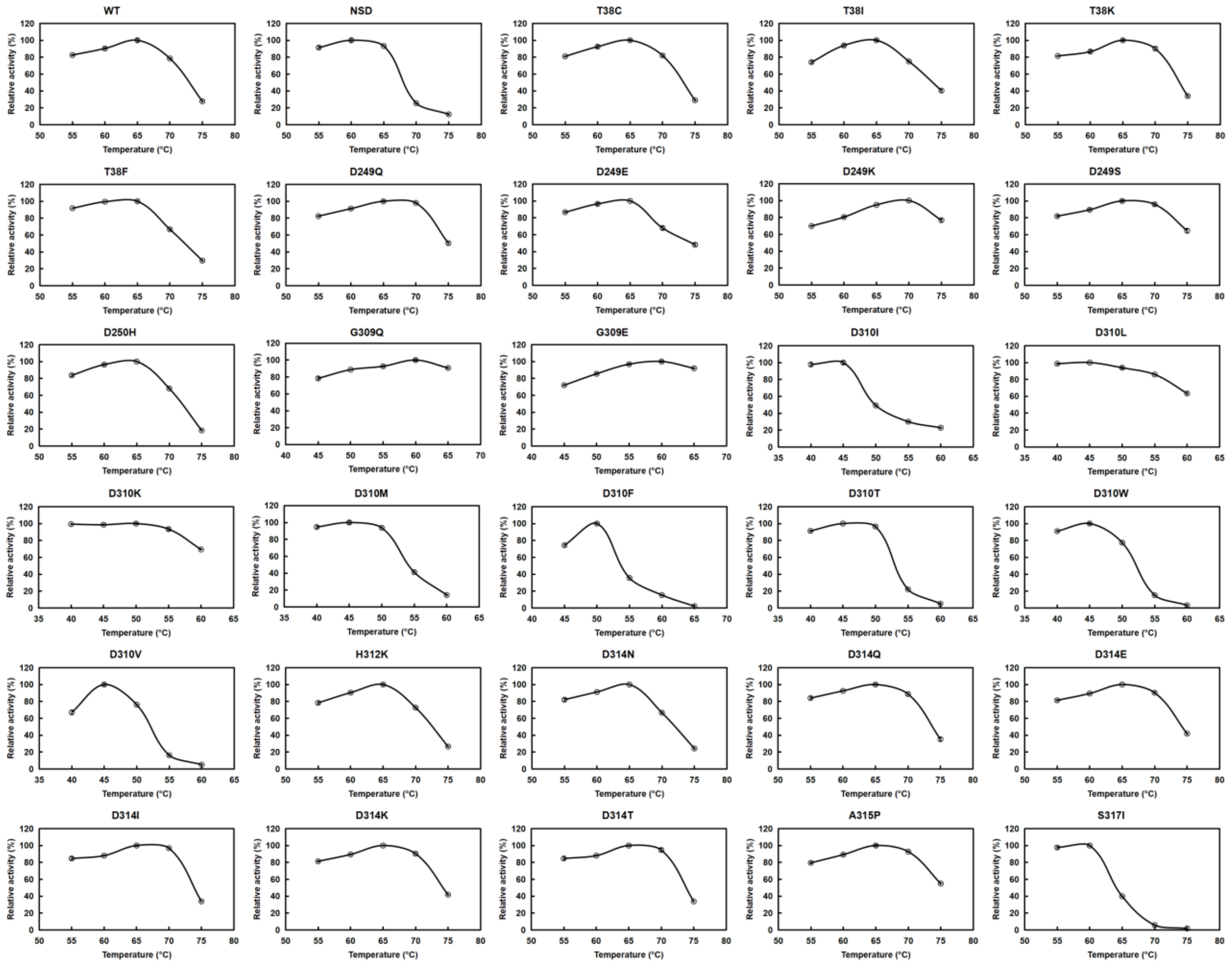

Figure S4: Effects of temperatures on the recombinant enzymes activities measured in Citrate-Phosphate (CPBS) buffer (pH 5.0) for 15 min.
